# Culturing intestinal stem cells: applications for colorectal cancer research

**DOI:** 10.3389/fgene.2014.00169

**Published:** 2014-06-05

**Authors:** Masayuki Fujii, Toshiro Sato

**Affiliations:** Department of Gastroenterology, School of Medicine, Keio UniversityTokyo, Japan

**Keywords:** cancer stem cells (CSC), wnt proteins, R-spondin, organoids, niche

## Abstract

Recent advance of sequencing technology has revealed genetic alterations in colorectal cancer (CRC). The biological function of recurrently mutated genes has been intensively investigated through mouse genetic models and CRC cell lines. Although these experimental models may not fully reflect biological traits of human intestinal epithelium, they provided insights into the understanding of intestinal stem cell self-renewal, leading to the development of novel human intestinal organoid culture system. Intestinal organoid culture enabled to expand normal or tumor epithelial cells *in vitro* retaining their stem cell self-renewal and multiple differentiation. Gene manipulation of these cultured cells may provide an attractive tool for investigating genetic events involved in colorectal carcinogenesis.

## INTRODUCTION

Despite recent advances in therapeutics, colorectal cancer (CRC) is a major health issue; more than a million people develop CRC, causing more than 700 thousand deaths worldwide yearly (Lozano et al., 2012). Surgically non-resectable tumors or metastatic disease ultimately acquires resistance to therapy, leading to death (Cunningham et al., 2010). The notion that a limited number of cells within a cancer are exclusively capable of initiating and maintaining the tumor, i.e., the cancer stem cell (CSC) hypothesis, has recently been gaining favor, and CRC is no exception. CSCs are referred to as being resistant to therapy, responsible for tumor metastasis and recurrence, and potential targets of new therapeutic strategies. Investigators have attempted to identify or isolate colorectal CSCs; however, direct evidence of colorectal CSCs has been lacking to date (Clevers, 2011).

Recently, crypt base columnar cells (CBC cells) lying at the bottom of intestinal crypts were shown to give rise to all lineages of intestinal epithelial cells by genetic tracing of the *Lgr5* gene (Barker et al., 2007). Genetic transformation of these Lgr5^+^ intestinal stem cells (ISCs) has shown their potential as tumor-initiating cells (Barker et al., 2009). A method of maintaining and expanding ISCs *ex vivo* has also been established (Sato et al., 2009). This dramatic progress has provided new insight into ISC biology and may prove useful in understanding the relationship between ISCs and colorectal CSCs.

## IDENTIFICATION OF INTESTINAL EPITHELIUM STEM CELLS

The intestinal epithelium is one of the most rapidly renewing tissues in the adult mammalian body, with complete turnover every 4–5 days (Barker et al., 2008). The small intestine epithelium comprises two histologically distinct structures: the villi projected toward the gut lumen, and the crypts invaginating into the mucosa. The villus contains three types of post-mitotic differentiated intestinal cells with the divergent functions of absorption (enterocytes), mucus secretion (goblet cells), and hormone secretion (endocrine cells). Paneth cells, which secrete lysozyme, reside at the base of the crypt. The colorectal epithelium lacks villi and Paneth cells, although the general structure remains similar to that of the small intestine.

The existence of long-lived ISCs capable of generating all other types of intestinal cells was first proposed by Stevens and Leblond (1947). Their pulse-chase analysis of ^3^H thymidine-labeled proliferating cells by autoradiography demonstrated that continuously proliferating intestinal crypt cells completely replace the villus cells every 3 days. This finding later led to the concept that all differentiated intestinal cell types ultimately originate from undifferentiated cells residing at the bottom of the crypt, specifically, the crypt base columnar cells interspersed between the Paneth cells (Cheng and Leblond, 1974).

Subsequent work by Potten et al. (1978) found that CBCs residing at position +4 relative to the crypt bottom retained the radio-isotopic DNA label, suggesting that these cells were very slowly dividing or quiescent. Because tissue stem cells were thought to be relatively dormant to evade DNA damage or telomere shortening during DNA replication, these findings led later investigators to assume that +4 position “label-retaining cells” were the ISCs.

**FIGURE 1:**
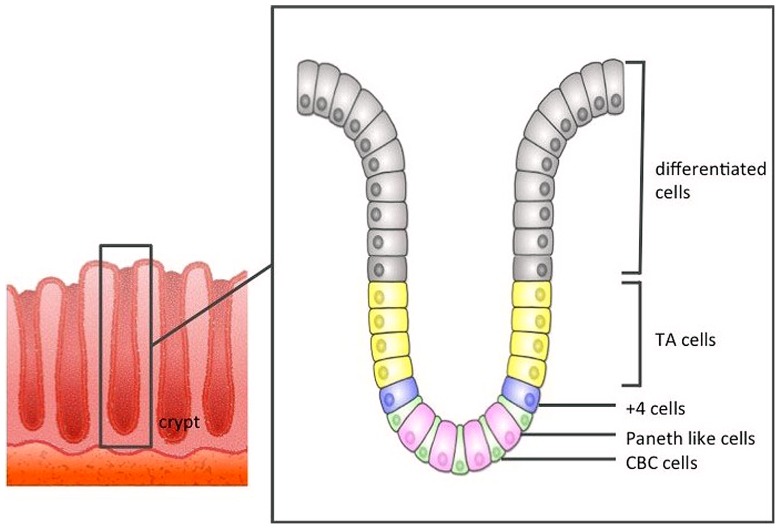
**The structure of the large intestine crypt**. Each crypt comprises crypt base columnar cells (CBC cells) at the bottom, and these CBC cells are driven toward the lumen as they differentiate.

Direct evidence that CBCs were in fact ISCs remained elusive until 2007, when Barker et al. (2007) using an *Lgr5-EGFP-IRES-creER*^T2^ knock-in transgenic mouse lineage tracing approach, reported that CBC cells exclusively express the *Lgr5* gene, and these Lgr5^+^ CBCs generated all types of differentiated intestinal epithelial cells. Lgr5^+^ stem cells divide every 24 h, giving rise to progeny called “transit-amplifying cells” (TA cells) that reside just above the crypt stem cell zone. TA cells divide vigorously, generating 16–32 differentiated cells daily. Differentiated epithelial cells are pushed out along the crypt–villus axis toward the tip of the villus, before eventually being sloughed off into the gut lumen 4–5 days later (**Figure 1**).

Similar lineage tracing studies using genes expressed in quiescent +4 cells (*Bmi1*, *mTERT*, *HOPX*, and *Lrig1*) have shown that these cells can also yield all intestinal epithelial lineages (Sangiorgi and Capecchi, 2008; Montgomery et al., 2011; Takeda et al., 2011; Powell et al., 2012). This led to the idea that quiescent +4 cells may revert back to robustly dividing Lgr5^+^ stem cells upon crypt damage, thus acting as an ISC reservoir (Tian et al., 2011). Although this explanation may account for the co-existence of active Lgr5^+^ cells and quiescent +4 cells with ISC capabilities, it has since been shown that genes expressed in +4 cells are also expressed in Lgr5^+^ cells and differentiated intestinal cells (Munoz et al., 2012).

Interestingly, secretory progenitor cells that redundantly express the Notch ligand delta-like1 (Dll1) have been shown to revert to Lgr5^+^ stem cells upon intestinal damage (van Es et al., 2012). More recently, a fraction of Lgr5^+^ cells were identified as the label-retaining cells (LRC) and were shown to be committed to differentiate into Paneth cells (Buczacki et al., 2013). Buczacki et al. (2013) using an elegant lineage tracing strategy, demonstrated that these Lgr5^+^ LRCs formed clonal crypt structure after intestinal damage. Although Lgr5^+^ LRCs and Dll1^high^ cells are not identical in location or Lgr5 expression, these studies indicate plasticity between the secretory progenitors and ISCs and that reserve pools may exist that can regain stem cell signatures upon crypt damage.

## CELLS OF ORIGIN IN COLORECTAL NEOPLASMS

**FIGURE 2:**
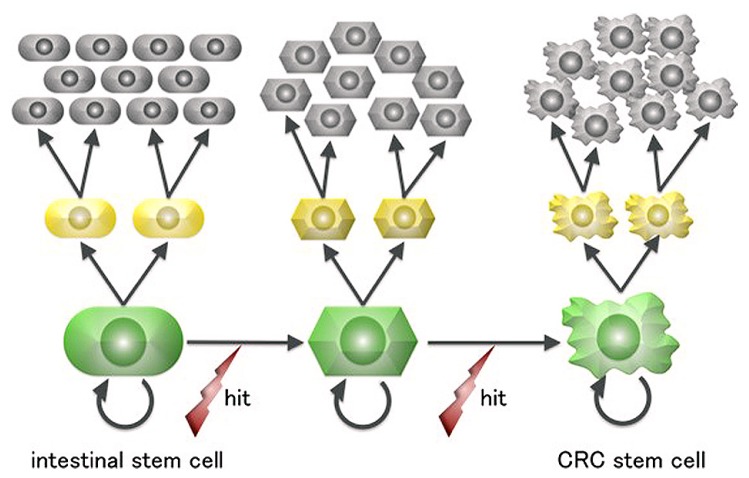
**A scheme for colorectal cancer stem cell generation from intestinal stem cells**. Intestinal cells are the only long-lived cells in the human large intestine epithelium and thus can undergo the multiple mutagenic events required for neoplastic transformation.

Terminally differentiated intestinal cells are post-mitotic and have a lifespan of 4–5 days before being shed into the gut. This short-lived fate is irreversible and renders it unlikely that they would accumulate a sufficient number of “driver” mutations for neoplastic growth, especially considering that mutagenesis in human cells is a rare event (Drake et al., 1998). In contrast, ISCs are the only long-living cells in the intestinal epithelium and are thus more plausible candidate cells of origin for intestinal tumors (**Figure 2**).

Indeed, recent studies have demonstrated that Lgr5^+^ cells may also function as stem cells within intestinal adenomas (Barker et al., 2009). Barker et al. (2009) crossed *Lgr5-EGFP-IRES-creER*^T2^ knock-in mice with *APC*^flox/flox^ mice to produce an Lgr5^+^ stem cell-specific knockout of *APC*, which resulted in the formation of macroscopic adenomas. In contrast, upon deletion of *APC* in TA or differentiated intestinal cells, these cells only formed microscopic adenomas. These data suggest that Lgr5^+^ stem cells, but not their differentiated progeny, are potential cells of origin of intestinal adenoma.

## IDENTIFYING COLORECTAL CANCER STEM CELLS

Cancer stem cells are defined as the cancer cells that drive tumorigenesis through long-term self-renewal and production of differentiated, non-tumorigenic progenies. The present gold standard for defining CSC “stemness” is to show their capacity to transfer disease into immunodeficient mice at limiting dilutions. This xenograft assay involves fluorescence-activated cell sorting (FACS) of single cancer cells that exhibit the putative CSC cell signature and subsequent quantification of their ability to develop tumors resembling the original tumor. While this assay represents the only methodology presently available, it is important to consider its limitations when interpreting the resultant data. First, the CSC markers that have been used to date only enrich, to various degrees, the CSC fraction within the population; they do not permit complete discrimination between the CSC and non-CSC pools. Second, differences between the tumor microenvironment of the original site and the transplanted recipient may impact CSC function (Bissell and Labarge, 2005). Growth factors or hormones essential for the tumor growth may be absent, or growth may be attenuated due to the species barrier between rodents and humans.

A major focus for CSC research has been the identification of surrogate markers that distinguish CSCs from non-CSCs within the tumor bulk. With respect to CRC, prominin-1 (CD133) was initially used as a putative CRC stem cell marker. CD133-positive cells derived from human CRCs generated tumors histologically identical to the original tumors in the xenograft assay, whereas CD133-negative cells showed reduced tumor initiation (O’Brien et al., 2007). However, this finding was contested by other studies demonstrating that CD133-negative cells propagated tumors as well (Dalerba et al., 2007; Shmelkov et al., 2008). Sorting by other surface markers, such as CD44 (Dalerba et al., 2007), CD166 (Levin et al., 2010), and ALDH1 (Huang et al., 2009), and by a combination of such markers was employed to isolate CRC stem cells in later studies. More specific markers of ISCs, such as LGR5 or EPHB2, have also been reported to mark the CRC stem cell population (Merlos-Suarez et al., 2011; Kemper et al., 2012). More recently, Schepers et al. (2012) demonstrated genetic lineage tracing of Lgr5^+^ cells within mouse adenomas, indicating that a small population of cells within the adenoma (5–10%) was responsible for adenoma self-renewal and production of differentiated Lgr5^-^ adenoma cells. Compared with FACS-based experiments, in which cells are detached from the niche and dissociated into single cells, genetic lineage tracing experiments might provide more physiological results. Genetic tracing experiments using human CRC samples are warranted in future studies.

## GENETIC ALTERATIONS IN CRC

Colorectal tumors can be stratified into a number of groups based on their mutational profile, which suggests several distinct routes of colorectal neoplastic formation are possible. One well-established pathway is the multistep genetic carcinogenesis initially proposed by Fearon and Vogelstein (1990). This pathway is referred to as the adenoma to carcinoma sequence, as CRCs arising via this pathway originate from tubular adenomas. In particular, this pathway is triggered by *APC* gene inactivation, which results in ligand-independent Wnt pathway activation, followed by genetic aberrations in various signaling pathways such as *KRAS* in RAS–RAF pathway, *SMAD4* in transforming growth factor beta pathway, *PIK3CA* in AKT-mTOR pathway, and *TP53*. These types of CRCs almost invariably accompany chromosomal aneuploidy or instability of the genome characterized as chromosomal instability (CIN).

Shortly after the proposal of a multistep model for CRC carcinogenesis, subsets of CRCs were shown to carry shorter repetitive DNA elements or microsatellites than normal tissues (Ionov et al., 1993). This signature, microsatellite instability (MSI), marks impairment of the DNA mismatch repair (MMR) system and is observed in CRCs from Lynch syndrome or so called hereditary non-polyposis colon cancer (HNPCC) patients (Peltomaki et al., 1993), as well as in 12–17% of the sporadic CRCs (Ward et al., 2001; Popat et al., 2005). These sporadic CRCs with MSI exhibit clearly different molecular signatures from CIN CRCs: they are near-euploidy or chromosomally stable and are associated with the *BRAF* gene mutation (Rajagopalan et al., 2002). Epigenetic silencing of the MMR genes, mainly *hMLH1*, is often observed (Kane et al., 1997). Further investigations have shown that not only *hMLH1* but also numerous other genes comprising CpG dinucleotide-rich promoter regions are predisposed to epigenetic silencing by promoter methylation (termed the CpG island methylated phenotype, CIMP; Toyota et al., 1999). Serrated polyps of the colon, predominantly microvesicular hyperplastic polyps (MVHPs) and sessile serrated adenoma/polyps (SSA/Ps) were later found to exhibit molecular features similar to those of MSI CRCs (Yang et al., 2004), indicating their potential as the precursors of MSI CRCs. This pathway is referred to as the serrated pathway, arising from serrated polyps to sporadic CRCs with MSI, successively acquiring the *BRAF* mutation, CIMP, and MSI along with tumor development.

Another pathway of colorectal carcinogenesis, the alternative pathway arising via traditional serrated adenomas (TSAs) has also been proposed (Shen et al., 2007). This pathway is associated with *KRAS* mutation, *MGMT* (O^6^-methylguanine-DNA methyltransferase) methylation and MSI (Ogino et al., 2007), although the molecular details of this pathway remain elusive.

Recent large-scale sequencing analyses have identified recurrently mutated genes in CRCs. The initial report by Wood et al. (2007) demonstrated that approximately 80 genes are mutated in a typical CRC; however, most of these are neutral, “passenger,” mutations, and not more than 15 mutations are responsible for the initiation, progression, or maintenance of the tumor, i.e., are “driver” mutations. The extensive genetic analysis conducted by the Cancer Genome Atlas project identified the frequency and patterns of altered signaling pathways in sporadic CRCs (Cancer Genome Atlas, 2012). In this report, the cases were classified into two subtypes, non-hypermutated tumors (with a low frequency of gene mutations) and hypermutated tumors (with a high mutation frequency), roughly corresponding to CIN CRCs and MSI CRCs, respectively. Each subtype showed a disparate pattern of genetic mutations, supporting the idea that they arise from discrete pathways from which they arise.

## EXPANSION OF INTESTINAL STEM CELLS *EX VIVO*

The long-term culture of non-transformed intestinal cells has previously been unachievable until we established a method that enabled the expansion of murine ISCs *ex vivo* for more than a year (Sato et al., 2009). This method requires laminin-rich Matrigel to provide the cells with scaffolds, along with culture medium containing the growth factors and the hormones necessary to maintain ISCs: R-spondin 1; EGF; and Noggin. R-spondin 1 was later identified as the ligand for Lgr5 and essential for the effective activation of the Wnt signal (Carmon et al., 2011). EGF is associated with intestinal proliferation, and Noggin negatively regulates the BMP signal, which induces crypt differentiation. Under such conditions, ISCs give rise to additional Lgr5^+^ cells as well as differentiated intestinal cells and build three-dimensional cystic crypt–villus structures (organoids), reminiscent of the *in vivo* intestinal epithelium. Lgr5^+^ cells and Paneth cells reside at the bottom of the crypt component, whereas the villus component comprises differentiated intestinal cells. These organoids can be grown from a single sorted Lgr5^+^ stem cell by addition of the Rho kinase inhibitor, confirming the “stemness” of the Lgr5^+^ cells. We later demonstrated that this method could also be applied to human ISCs as well as human colorectal adenomas and adenocarcinomas with modification of the medium content (Sato et al., 2011). The culture medium for human colorectal stem cells requires Wnt3a, a p38 inhibitor and an ALK 4/5/7 inhibitor in addition to the murine small intestine culture condition, while the colorectal tumor organoids can grow in the absence of certain growth factors, depending on the pathway mutations they harbor. Most organoids derived from colorectal neoplasms can grow after the withdrawal of Wnt3a and R-spondin1, consistent with their *APC* mutation. Alternatively, *KRAS* mutation in the organoids renders EGF dispensable.

## APPLICATION OF ORGANOIDS TO CSC STUDY AND PERSPECTIVES

A forward genetic approach is essential for functional analysis of the candidate genes involved in CRC stem cell development from ISCs. Genetically engineered mice such as *Lgr5-EGFP-IRES-creER*^T2^/APC^flox/flox^ mice allow the *in situ* observation of tumor generation from normal intestinal epithelium and the kinetics of the stem cells within the tumor (Barker et al., 2009). However, the establishment of genetically engineered strains requires substantial time, effort and cost, especially when handling multiple genes. Clearly, similar approaches in humans are not possible, with the very rare exceptions of patients with certain inherited disorders.

Organoids are amenable to gene overexpression or knockdown by viral infection (Koo et al., 2012), which provide a unique tool to study the phenotypes resulting from the manipulation of gene expression in human ISCs. Furthermore, Schwank et al. (2013) recently demonstrated the application of the CRISPR/Cas9 system for genome targeting in organoids. In this report, the cystic fibrosis transmembrane conductor receptor (CFTR) gene of the intestinal organoids, derived from cystic fibrosis patients, was corrected by homologous recombination via the CRISPR/Cas9 system. A similar methodology can be employed in the context of oncogenes or tumor suppressor genes as well. In summary, the ability to use organoid culture to model the genetic alterations associated with CRC carcinogenesis provides a promising method by which the genetic events involved in CRC stem cell generation can be functionally studied.

## Conflict of Interest Statement

The authors declare that the research was conducted in the absence of any commercial or financial relationships that could be construed as a potential conflict of interest.
